# Book Reviews

**Published:** 1815-01

**Authors:** 


### CRITICAL ANALYSIS

### OF RECENT PUBLICATIONS

IN THE

### DIFFERENT BRANCHES OF PHYSIC, SURGERY, AND MEDICAL PHILOSOPHY.

---

*Pathological Researches.—Essay I. On Malformations of the Human Heart: illustrated by numerous Cases, and five Plates, containing fourteen Figures, and preceded by some Observations on the Method of improving the Diagnostic Part of Medicine.* By J. R. FARRE, M.D. 8vo. pp. 46.

*(Continued from p. 517.)*

WE resume with pleasure our notice of this valuable work. The next variety which the author describes is "I. 2. *d. Ostium Arteriæ Pulmonalis communicating with both Ventricles.*"—This is a rare malformation; the cases of it detailed by Dr. Farre, are furnished by Mr. A. Cooper and Mr. English. Two plates illustrate the disease.

"I. 2. *e. Dilated Foramen Ovale, and contracted Ostium Arteriæ Pulmonalis.*"—Of this variety the author only notices two cases: one of them is related in the 17th Letter of Morgagni, art. 12, 13; the other in the 6th vol. of the Commentaries of the Academy of Sciences at Bologna, and is also translated in the 6th vol. of the London Medical Journal.

"I. 2. *f. Dilated Foramen Ovale, with an Open Ductus Arteriosus, and Impervious Ostium Arteriæ Pulmonalis.*"—Although this variety is more frequent, the author has not alluded to any case in his own practice. He refers, however, to one published by Dr. W. Hunter in the Medical Observations and Inquiries, vol. vi. p. 291, and by Mr. Hodgson, in the 5th vol. of the London Medical Review.

"I. 2. *g. Perforation of the Septum Ventriculorum.*"—Described by Dr. W. Hunter and Corvisart.

"I. 2. *h. Ostium Aortæ communicating with both Ventricles.*"—This variety is not very rare; the author has enumerated several cases of it from Abernethy, Cooper, Corvisart, and other writers. The only case which he details from his own observation is the following.

"A female infant, four weeks old, was brought to me at the London Dispensary, Artillery-street, in the month of April 1809. The colour of her skin was blue, and as often as she cried, which happened whenever she was moved, the colour became very dark. Being well clothed, her skin was of a good warmth; but her mother assured me that she chilled fast, and that it was very difficult to keep keep her warm. Her respiration was short, but not very laborious. The umbilicus was ulcerated, and the surrounding integuments were inflamed. Under one of her arms, an ulceration of the skin, tending to gangrene, had taken place. The functions of the alimentary canal were badly performed, and her feeble powers were wasted by a diarrhœa. She lived only a week longer.

"*Dissection.*—The septum ventriculorum was perforated; the aorta arose from both ventricles, and was dilated; the pulmonary artery was imperforate, and as far as its bifurcation, and its branches received their blood from the aorta through the ductus arteriosus. The right auricle and ventricle were larger than the left."

A neat figure gives a tolerable representation of this variety of imperfect structure.

"I. 2. 1. *Transposition of the Aorta and Pulmonary Artery.*"—Several cases of this singular variety are related, and this portion of the essay concludes with some observations on the symptoms of malformations of the heart, and on the temperature of persons with malformed hearts, that will be perused with peculiar interest. The subjoined extracts will fully evince the author's talent for observation, and demonstrate the utility of his investigation.

"1. *A permanent blue colour of the skin.*—The blue approaches to a black colour in proportion to the diminished size of the pulmonary artery, and of the ductus arteriosus. It affords a sign of the first species of malformation of the heart, or that which mingles black with red blood, being present in a very large proportion of these cases; but it has already been proved, that malformation may exist although this sign be not present. See I. 1. *a.* and *c.* Another exception occurs in the second example of the variety, I. 2. *d.* The patient's skin was always very pallid. From these exceptions we learn, that if the *full* proportion of blood be circulated through the lungs, although the red blood be subsequently mixed with an equal proportion of black blood in the heart, yet a black colour will not be imparted to the skin. The fact, at present, applies only to the infant. Evidence is wanting to prove its application to the youthful and the adult state.

"In difficult transmission of blood through the heart, or its vessels, especially its pulmonary branches, the skin is pallid, transiently livid, of a violet colour, or permanently blue, according as its capillary vessels are more or less completely filled, but chiefly in proportion as the circulation is retarded through the lungs. The same effects are produced by whatever diminishes the capacity of the air cells in a remarkable degree. It is certain, however, that the peculiar blue colour of the skin, which has even given a name to those who have malformed hearts, is far more characteristic of mingled black and red blood, the former being in excess, than it is of an impeded circulation, and constitutes the most material part of the diagnosis.

"2. *Cold.*

"2. *Coldness of the Skin.*—This sign naturally follows the blue colour of the skin, as both are produced by an over proportion of black blood. It may be worth the trouble of reconciling the contradictory evidence respecting this sign, by tracing the variation in the structure, by which the difference of temperature may be justly explained. If the reader will examine *d.* and *e.* in figure 3, and compare them with *b.* and *g.* in figures 12, 13, 14, he will perceive that signs of a diminished temperature at the surface of the body were manifested in proportion as the pulmonary artery, or, in the event of the obliteration of that artery, the ductus arteriosus, diminished in size; and that it was sensibly least in the example in which the ductus arteriosus was smallest. If, in I. 1. *b.* and in the second example of I. 2. *f.* the observations respecting the external temperature of the body be correct, it would appear that there is an over proportion of black blood, which gives the first sign—a permanent blue colour, without manifesting, in the infant state, the second sign—a coldness of the skin. Although this observation needs farther proof, it is certain, that a still greater excess in the proportion of black blood, as in some of the cases above referred to, affords both signs in a striking degree. The evidence of this fact will also be found in all the examples of I. 2. *i.*

"3. *Paroxysms of irregular respiration—of screaming—of panting. Respiration, remarkably quick—continually difficult or laborious—sense of suffocation—cough.* The disturbed state of the breathing by paroxysms, seems to be more characteristic of mingled black and red blood; but the dyspnœa, of difficult transmission of blood through the heart and its great vessels.

"4. *Palpitation—vehement action of the heart—pulse irregular, quick, and weak—intermittent. Serous effusion* into the cavities and cellular membrane, manifested by œdematous legs and bloated face. *Hæmorrhages* from the nose, gums, lungs, &c. These signs, varying in degree, and more or less combined, are found in many cases of malformation, and are indicative only of an impeded circulation.

"5. *Torpor of the Brain—Epilepsy—Apoplexy—Paralysis—Syncope.*—These effects are to be attributed partly to an impeded circulation through the heart, to the accumulation of blood in the brain, and the consequent pressure sustained by that organ, and partly to the want of those renovating powers which red blood may impart to the brain.

"6. *Defective Nutrition.* This effect varies in degree from the slender to the emaciated figure, according to the condition of the alimentary canal and its dependant organs; for in some there is too little appetite, in others too much;—in some, constipation; in others, diarrhœa. But, if the natural functions be tolerably well performed, and the due proportion of chyle absorbed, it is not unreasonable to expect an accumulation of adipose matter in a subject whose circulation is disturbed or impeded, whose blood cannot always warm the surface of his body, and whose nervous system is without energy. Such was the actual condition of the boy whose case was recorded by Dr. Richard Pulteney, and whose figure must have been the reverse of that which Dr. Hunter, in a peculiar but forcible manner, thus described: 'If a man had never seen any of the canine species but the bull-dog, for example, he would be much struck at the first sight of the slender and delicate Italian greyhound. This young gentleman's figure put me in mind of that animal; and, when I looked upon his legs particularly, I could not but think of the limbs of a wading water-fowl.'

"The *almost sudden* extinction of life in man, and other warm-blooded animals, by the various accidents which suspend respiration, is not fully explained by saying that the source of heat is cut off, and the excretion of a matter deleterious to the system is interrupted. Other circumstances contribute to that event, the most important of which, it is probable, remains unknown; but there is one, which, without insisting too strongly on its importance, is yet very worthy of being noticed, because it may serve to guide our judgment in cases of malformation of the heart, where the diagnosis becomes more than usually difficult. I mean the association which exists between the action of the heart, and the action of the muscles of respiration. The suspension of the latter must materially derange the former. But these associated motions are reciprocal, so that any remarkable imperfection in the functions of the heart will also be generally attended with a disordered respiration: thus, in I. 1. *a.* and in the third case of I. 2. *c:* in which neither a blue colour, nor a coldness of the skin, were remarkable, the peculiarities of their respiration marked the malformation of their hearts.

"Finally, on the subject of mingled black and red blood, it is expedient to direct the attention of the profession to the following desiderata:—1st, The accurate measurement, by the thermometer, of the *internal* and external temperature of the patients. The former may be done by placing the bulb of the thermometer under the tongue, or, more conveniently in infants, by introducing it into the rectum, as Dr. Baillie long ago suggested. In the cases which have already been communicated to the profession, as well as in some of those which are here added, we have, as it were, with one consent, neglected this important experiment. In one instance, however, of a lady whose skin suddenly became and remained permanently blue, from a lesion, as it was supposed, of the septum of the heart, Mr. Astley Cooper ascertained that her internal temperature was 100 degrees, whilst her extremities were sensibly colder than natural. This interesting observation supports the result of the experimental inquiry on this point by Mr. Coleman, which he published in his valuable work on Suspended Respiration, and invites farther investigation. 2dly, The quantity of carbonic acid gas which may be formed by the subject of a malformed heart, during successive acts of respiration in a determined period, compared with the quantity formed by a perfect subject of like age and figure.

This

This experiment will be far more difficult than the former, and its result must be more dubious; but, if it can be made with any tolerable approximation to the truth, it is expected that it will furnish an interesting fact, especially in those patients whose skins are permanently blue and easily chilled."

We have now to notice the second species, or MALFORMATIONS OF THE HEART, OR OF ITS ARTERIES, ONLY IMPEDING THE CIRCULATION OF THE BLOOD.

"II. *a. Left Ostium Ventriculi contracted, and Mitral Valve rigid.*"—The author is not quite decided whether to assign such appearances to malformation, or to disease. Mr. A. Burns has described the variety as a species of malformation, and Dr. Farre, who does not seem fond of forming his judgment on slight grounds, seems inclined to admit this opinion, until he obtains further evidence on the subject. The case related by Dr. F. was published, with some others, by Mr. Burns, in his treatise on Diseases of the Heart.

"II. *c. Ostium Aortæ narrowed by having two instead of three Semilunar Valves.*"—This variety is very uncommon; we have therefore pleasure in transcribing the following interesting case of it.

"Mrs.———, aged 24, a delicate woman, of the middle size, eight months married, in the eighth month of her pregnancy, died suddenly on Wednesday morning, the 27th of May, 1812. Mr. Saner, who resided on the spot, instantly attended. He found her countenance suffused and very livid, but she was already dead. On the 28th, twenty hours after her death, Mr. Saner favoured me with the opportunity of conducting the dissection, which was done with the assistance of himself and Mr. J. Burrows.

"The brain, minutely examined, presented only the natural appearances. The longitudinal and lateral sinuses of the dura mater were rather more turgid with fluid blood than they usually are.

"The heart had the parietes of its left ventricle much thickened, and the valves of its aorta malformed. Two, instead of three semilunar valves, had been formed, and these were so united as to constitute one membrane, having an oval aperture in its centre. Ossific matter was deposited on the surface of the valve next to the ventricle. A moderate quantity of serum, tinged with red particles, was found on both sides of the chest. The lungs were sound.

"The viscera of the abdomen were in a natural state. The uterus had contracted just below the umbilicus: to its internal surface the placenta and membranes yet adhered. The fœtus had been turned and extracted by Mr. Saner immediately after the mother's death. Although this humane effort to preserve its life was not crowned with success, the circumstances of the case being unfavourable, yet the attempt merits praise. Such an effort is not incompatible even with a trial of means for restoring animation to the the mother. The Cæsarian section, for the preservation of the infant, ought only to be used when the mother has suffered a violent death.

"I was informed, by her husband, that he had been acquainted with her only twelve months previous to their marriage. During that period she could never bear quick motion. Since marriage, he had observed, besides a difficulty in her breathing, frequent palpitation: he could even hear her heart beat as he lay beside her. The dyspnœa had so much increased during her pregnancy, that at night he had been obliged to carry her up-stairs to bed. Mr. Smart informed me that he had attended her for symptoms of pneumonia, a few months before her death; but he did not, at that time, remark any peculiarity in her pulse."

The essay terminates with some short but feeling remarks on diminishing the sufferings of patients in cases of malformation where the cure is hopeless. We have little doubt that, as the author's experience accumulates, he will be enabled to arrive at some degree of certainty in what he now proposes as hints rather than as axioms. The chief means in our power, at present, are temperature and posture; both of which in some cases may be so regulated as to lessen pain and prolong life. The second and third essays, which we hope will shortly come before the public, will treat of inflammation of the heart, both in the acute and chronic form, when the author will find a more ample field for considering the effects of difficult transmission of blood through the organ.

---

*The Morbid Anatomy of the Brain in Mania and Hydrophobia, with the Pathology of these two Diseases, as collected from the Papers of the late Andrew Marshal, M.D. many Years Teacher of Anatomy in London; with an Account of some Experiments to ascertain whether the Pericardium and Ventricles of the Brain contain Matter in a State of Health: to which is prefixed, a Sketch of his Life.* By S. Sawrey, Member of the Royal College of Surgeons, formerly Assistant-Lecturer to Dr. Marshal.—8vo. pp. 294. Longman and Co. London.

The memoirs of every public character are interesting. Those presented to us of Dr. Marshal are particularly so, as they describe a character exactly similar to his external appearance and habits. The first paragraph of Mr. Sawrey expresses this much better than we can do.

"The life of Dr. Marshal does not present any of those incidents which give interest to biography. It had no romantic adventures, nor was it checquered by any singular misfortune; it was was the life of a man of original genius, emerging, unassisted, from his native obscurity, quietly and unostentatiously maturing himself by studies and meditations unknown to the world: long hesitating in the choice of a profession; beginning late, but pursuing it, when decided upon, with all that force of mind and enthusiasm which make difficulties but the means of increased progress. At all times rather shrinking and secluding himself from public notice, than ambitious of notoriety, and yet calmly and steadily advancing to knowledge and reputation; so that he was enabled to meet the decline of life with a competence satisfying all his wishes, and with high professional respectability."

Dr. Marshal, it appears, was the son of a farmer in Fifeshire, and seems always to have been passionately fond of rural scenery. Probably he would have become a practical farmer, but that, like Burns, he soon found the impracticability of struggling against oppressive rents in a country which requires constant labour. The gravity of his temper was, perhaps, the only reason why he pursued a more even tenor; and by dint of industry and economy procured a very moderate independence in the decline of life.

"Merely," says he, as appears by his own MS. "from seeing the flocks and herds of my father, and the beautiful scenes of a rural life and business, I remember to have said in a moment that I would be a farmer, and give up school. My father immediately complied with my wish, but as I was only fourteen, he sent me a winter more to school to learn arithmetic. I deceived myself in this too, for I learned very little. My old Latin master was dead; a new one had succeeded, but his method was not the best.

"I returned home from Abernethy, and soon began to be a young farmer, working, ordering, &c. I took an infinite interest in some of the individuals of our cattle. I had great pleasure in seeing the mare and foal put on good grass, and the calves well fed, &c. But my father being obliged to give up his goods, from misfortune, when I was about sixteen, I became tired of farming, hurt at the disaster I saw befalling my father's interest.

"I resumed the study of Latin, and applied to it with some attention, being resolved to be a seceding minister; and feeling myself gifted in the ability of saying grace and prayer.

"I studied Latin again in Mr. Buchanan's school in Cairny-hill, and went thence to be examined at Culfargie and to be passed as a student of philosophy at Abernethy. I passed, and studied logic there under Mr. Pine. The logic was an old MSS. of St. Andrew's system. I learnt every word of it by heart, and became master of genus, species, syllogism, &c. I received more benefit from this winter than I have ever done from any other learning.

"There was another examination for students of divinity at Alloa. The first examination was in Latin, in Virgil, Book II. of the Æneid; the next was in practical religion. I gave an account of my conversion.

"About this time I taught school at Limekilns. I was charmed with the romantic scenes of nature, and was always enjoying them in solitude. I was ignorant how to get or how to keep money.

"About this time I wrote an Essay on Ambition, which was published in the R—— Magazine, and an Essay on Composition, published in the British Magazine. In this last I animadverted on an expression of a seceding minister—'O now we conclude.' For publishing this essay I was summoned to the synod at Edinburgh, and excommunicated.

"All this time I was so struck with the appearances of Nature, that to get settled and provided for was a subject which never came into my head. I passed one year wandering along the banks of the Forth; another along those of the Tay; astonished at every new scene of the country; fond of reading; not perfect in the Latin, yet admiring its composition.

"Having been to Glasgow with my brother William, on his way to America, I returned, and was presented with a guinea at Greenock, from Mr. Simson, for pleasing him in prayer.

"I then formed a blind design to go to Glasgow, and to leave my father's and the Limekilns. There, with my sister Ann, I kept house, and taught school; attended College, particularly Dr. Reid; and I studied Greek privately. I was introduced to Dr. W., who took little notice of me, but took my money. I also studied mathematics.

"After two years residence there I went by Dr. Reid's recommendation to be tutor to a family in Islay. Before I set out I had a fever in Glasgow. The symptoms were, great alteration in sense, weakness, sickness, and bad dreams. On my way to Islay I was struck with the appearance of the west coast. I was entertained by an highland laird. After being settled I became unaccountably hoarse, and much weakened. After the fever my complexion became unduly vivid.

"I was at Islay four years; little to do; striving to instruct unmanageable children. I was out of the world—nearly out of existence. I read some Latin by dictionary and grammar; read eight books of Livy, and wrote sonnets—Dear Rocks of Islay! Here I used to wander in solitude, admiring the ocean, the beach, and the islands scattered around.

"By this time I was not less than twenty-five years old. I left Islay, and was, as before, without any rational prospect of a better situation. I went to Edinburgh with my pupil. I did my utmost to instruct him and to save his father's money. I carried him back in the summer.

"I returned and subsisted by reading Latin and Greek with the students, privately. I attended the physical classes from curiosity. In 1769, I attended Dr. H—, as a student of divinity, which was my profession at Glasgow too. I was of the Divinity Hall when I delivered two discourses,—both barren of knowledge. I gave also a discourse at the Divinity Hall of the Seceders. I got reputation at Edinburgh for Greek. I was regularly studying new and difficult subjects; never engaging myself."——

"In

"In 1770 we find him engaged in private correspondence with the present Dr. Young, who was afterwards appointed Professor of Greek in the University of Glasgow, on the Greek language. He was then twenty-eight years old; and at this period he was thus esteemed by his literary friend:—

"'Your correspondence, my dear sir, will be extremely agreeable, and the more so as we shall converse without restraint or ceremony. You need not to have told me you can be faithful; I was convinced of that before. It is written in large characters, not in your letter (for there the characters are but dwarful), but in your face, your actions, and I presume somewhere else, which it is often difficult now-a-days to see. I have no mind to turn panegyrist; it is but a poor trade; but therefore I say, gratis, you have that in you which I think I can love,'" &c.

"The letter containing these passages is dated July 19, 1770, and is addressed to Mr. Marshal, student, at Mrs. Martin's, in Lady Stair's Close, Lawn Mercat, Edinburgh.

"From another letter of Dr. Young's, dated August of the same year, we have some intimation of Mr. Marshal's studies at that time.

"'Your metaphysics upon time, place, associations, trees, ideas, and islands, have raised you several inches in my estimation. As far as I can judge they are perfectly orthodox, and consonous to right reason. But, to be serious, you have certainly gone the true way to work. There is no doubt but language has its principles, and the more any one understands of it the more reason he finds to be convinced that it is so. The terms 'caprice of language' flowed originally from a weak noddle. These very caprices have their principles, and principles not incapable of investigation,'" &c.

We shall hereafter make further remarks on Dr. Marshal's metaphysics.

After this it appears that Mr. M. made a tour on the Continent as travelling tutor with Lord Balgonie, eldest son of the Earl of Leven and Melville. Before this time he had turned his attention to medicine, but did not seriously undertake it till about the year 1776: that summer he studied botany in Scotland, and in the following year arrived in London, to have the advantage of attending the lectures of the two Hunters. In 1778 he was appointed, by the interest of his noble pupil, surgeon to the 88th regiment, in which station he acquitted himself with judgment, fidelity, and industry. During this time he procured his degree from Edinburgh, having sent in an inaugural dissertation, "De tuenda salute militum."—Why does not the present compilation contain Dr. M.'s practical opinions on so important a subject?

After the peace of 1783, the regiment being disbanded, Dr. Marshal settled in London, by the advice and under the auspices of Dr. David Pitcairn, at that time physician to

St. Bartholomew's Hospital. There was then no teacher of any description at that house, if we except the gratuitous lectures on surgery given by Mr. Pott. Dr. M. met with ample encouragement as an anatomist, but from the deficiency of his voice it was a long while before he ventured to lecture. Probably he never was conscious of this defect himself, but was restrained at first only from a certain backwardness at extempore speaking, which was likely to attend one who commenced teaching at so advanced a period of life. At this time it seems to have been his intention to have practised surgery. No town practitioner will be surprised that he altered his resolution: surgery by itself, without the advantage of a hospital, is a bold undertaking for a stranger. At that time too Dr. Pitcairn had not gained that eminence by which he might have secured the success of his friend in a different department of medicine; nor indeed would it have been quite decent to have patronised a stranger to the disadvantage of his colleagues at St. Bartholomew's. In 1788, therefore, Dr. Marshal produced his degree, and procured his licence from the London college. Dr. M. had scarcely established his school in the neighbourhood of St. Bartholomew's, when the medical men of that venerable institution began to think it high time to erect something like a school within its walls. They could not but feel the decline of their emoluments, by the diminished number of pupils who were driven to other hospitals for the convenience of their respective classes. About this time too, Mr. Abernethy having completed his apprenticeship under Mr. Blicke, and showing talents well qualified for a public teacher, was fixed upon at an early age as the first lecturer in a newly-erected theatre. The following is Mr. Sawrey's account of this event:

"Some time after this, it was proposed by some mutual friends, that Dr. Marshal should allow Mr. Abernethy, then a rising and aspiring young man, to join him; to which intimation Dr. Marshal remarked,—'This will reduce me to a situation worse by half than that which was held out to me at first. I have embarked all my resources, and committed my reputation on this business, and by my industry I have attained some footing in it; and now I am to divide what is hardly enough when entire, and which I have laboured hard to enjoy entire.'—Thus considering it, no one will be surprised to find that Dr. Marshal declined the proposed partnership."

We shall at present make no comments on this subject, reserving them for our general observations on the character of Dr. M. He continued to lecture till the year 1800, when ill health, we are told, and probably it might be added increase crease crease of professional engagements, induced him to decline. He continued his practice with some interruptions from want of health for thirteen years more, when his constitution gave way to repeated attacks of a complaint very common at that age, and more frequently, it has been remarked, attacking literary and sedentary people.

"His health," says his biographer, "became gradually, but perceptibly, weak and precarious, for several years before his death, so that his friends had frequent cause of alarm, before the attack of the disease which proved fatal. For some time he had a disturbance in the urinary organs; but in July, 1812, the symptoms became more urgent; the micturitions were frequently painful and peremptory, excited upon trivial occasions, especially at night, breaking and disturbing his sleep. These symptoms were accompanied with frequent attacks of fever, which, as the complaint advanced, became more and more frequent and violent, until the day of his death, which was the 4th of April, 1813.

"During the period of this most painful illness, which Dr. Marshal supported with the greatest patience and fortitude, he constantly prayed for death, and looked forwards to it as his only remedy. To wish him better only caused him grief.

"One evening, when he was very ill, he asked the Editor, seriously, what he thought of the event of his disease. The Editor observed, that those frequent accessions of fever had certainly weakened him very much, yet he had shewn a strength of constitution, by getting rid of them, that induced some hope that the disease might get into a milder state, so that at least he might live in ease and a considerable degree of comfort. To which he answered,—'My dear sir, you distress me.'"

Such is the epitome of the life of an honest and enlightened man, we may add of a gentleman, and a scholar. But, in the description of his character, we have more of the composed feelings of resignation than of the active figure. That Dr. Marshal was an industrious anatomist, and a faithful teacher, cannot be questioned. If he was not perfect, he did not share more than the common failings of human nature. That there was something austere in his character, appears, we conceive, in all the more important transactions of his London life. Who but Dr. M. ever had a difference with Dr. D. Pitcairn? Why should he object to join Mr. Abernethy, whose youth, genius, and certain introduction into the hospital, must have secured a large school, of which Dr. M. would have probably shared the emoluments longer than he was enabled to sustain his own? His quarrel with Mr. Hunter was, we believe, at a period when the organic infirmity of the latter had rendered him highly irritable. There were probably faults on both sides: but

 whatever whatever failings of temper Dr. Marshal might be charged with, large allowances should be made for want of health, for the necessity of looking for the means of subsistence at an advanced age, and even for that unshaken integrity which is rarely attended with an over compliant disposition. A review of his posthumous works will be given in our next Number.

---

*Medico-Chirurgical Transactions, published by the Medical and Chirurgical Society of London. Volume the Fifth.* 8vo. pp. 447.—Longman and Co. 1814.

THE volume opens with an account of some cases of Periodical Jactitation or Chorea, by Dr. Watt, of Glasgow. They differ in some respects from the complaint which is so well known by the name of St. Vitus's dance, although that term seems more apt for them, than for the affection which occasionally occurs in children in this country.

Dr Denmark has contributed a case of Abscess in the Brain, which, being short, we shall transcribe.

"John Baynes, aged eighteen years, was admitted a patient of mine, from his Majesty's ship Fylla, on the 8th of last August, for inflammation of the right ear, attended with purulent discharge, but without fever. In the course of a few days the discharge ceased, merely by the use of purgatives and some topical lotions. On the 13th he complained of acute lancinating pain, confined chiefly to the top of the head, with a hard pulse, at about 100, and other symptoms of pyrexia. He was bled to 16 ounces, and had a purgative draught.

"14th. He had epistaxis during the night; the head-ache was relieved, but not removed; the tonge furred.

"16th. The symptoms continuing, the bleeding and purgatives were repeated.

"17th. After shaving the head I discovered a puffy diffused swelling over the whole of the right parietal bone. He recollects having received a blow upon the same part of the head, upwards of twelve months ago, with a piece of wood, which merely stunned him, without producing further subsequent inconvenience; but thinks the discharge from the ear might be dated from that period. Bleeding repeated.

"18th. A restless night with occasional delirium; white tremulous tongue; sense of chilliness; skin preternaturally hot; pulse 110; and hard tumour of the scalp not much elevated, but perfectly circumscribed and puffy, with acute pain on the slightest pressure. I made an incision through the tumour, five inches in length, down to the bone, when a very small quantity of pus issued. Several enlarged arterial branches were divided, from which between 20 and 30 ounces of blood were extracted, with the immediate effect of reducing the pulse in frequency, rendering it soft, and the patient tranquil and rational.

"19th. He slept a good deal during the night; the countenance was improved, and the delirium lessened.

"20th. Had a return of restlessness; the pulse was 90 and strong, and the wound in the scalp painful, with scarcely any suppuration. A purgative was given, and a blister was applied to the neck.

"21st. Was seized with convulsions last night, and has at present paralysis of the left arm, and retraction of the right angle of the mouth, with, at times, mild delirium; in the evening the bowels were open, but in other respects he continued the same.

"22d. Passed a restless night, but without convulsions; pulse nearly natural; a very trifling aberration of intellect; complains of head-ach, and the tumefaction of the scalp appears more general, retaining the impression of the fingers. The pericranium is now evidently detached on each side of the incision. I made a crucial incision across the sagittal suture, and downwards nearly as far as that of the squamous. I found the pericranium separated upwards of two inches; the bone was white, but seemingly deprived of nourishment, as no blood appeared on scraping it. The left arm was still paralytic—I applied the trephine close to the sagittal suture, and found the dura mater covered with pus, which also flowed through the suture during the operation. The discharge was so highly fœtid, as to be noticed by the patient, and seemed to be occasioned by a carious state of the diplöe, which was somewhat black and much thickened. The operation was succeeded by a partial removal of the paralytic affection, and an alleviation of every bad symptom; in which state of amendment he continued until the 25th, when he was again attacked with fever, with full and strong pulse.—Had a bolus of jalap and calomel.

27th.—A restless night with occasional delirium; acute pain extending along the whole course of the spine, from the occiput to the sacrum; a thin purulent discharge, with a very unhealthy appearance of the dura mater.

"28th. Restless, with white tongue, hot skin, delirium, and torpid state of the bowels. Complains much of the pain in his back, especially on every attempt to move himself from the horizontal to the erect posture, when the pain is so acute as to make him scream out most violently: he describes it as darting from the sacrum to the back of the head. The bolus was repeated.

"29th. More sensible, but restless; cannot bear the smallest elevation of the trunk. An opiate was given at bed-time, and on the following day the purgative bolus was repeated.

"31st. Features shrunk; countenance lurid; lips purplish; eyes half closed and glossy; pulse small and indistinct, with low muttering. From this state he was roused in the course of the day by stimuli; but he relapsed into it, became delirious, was affected with with subsultus tendinum, and porracious vomiting; and in this way continued until the evening of the 3d of September, when he died.

"*Dissection.*—The pericranium was detached from the whole of the superior part of the right parietal bone. During the sawing through the skull on the right side, several ounces of thin pus and bloody serum gushed out. On lifting the skull, a considerable quantity of purulent matter was found lodged between it and the dura mater, chiefly over the right hemisphere, but also extending for a short way over the left. This matter was traced downwards to the petrous portion of the right temporal bone, between the dura mater and skull, and appeared to have issued from a small circular ulceration of about two lines in diameter, in that membrane, immediately over the posterior lobe of the right hemisphere. This was confirmed by making an horizontal section of that lobe, two inches below its surface, which discovered an ulcerated cavity, one inch and an half in diameter, with indurated parietes, full of pus, and communicating with the aperture above in the dura mater. The form of this ulcer being that of a cone, or inverted funnel, seems to show that the ulcerative process commenced in the brain. There was another ulcer posterior to this, but it affected merely the cortical substance of the brain, and had not corroded its membranes. These were posterior to the perforation made by the trephine, through which the matter had free egress. On lifting the dura mater from the brain, there were adhesions, and a great quantity of pus between the right hemisphere and falciform process. This was accounted for by supposing it to be a part of that secreted in the ulcers before described, which, instead of passing through the eroded part of the dura mater, had pervaded the easier course between that membrane and the pia mater. The ventricles were enlarged, and filled with serum, which, from the appearance of these cavities, and of the plexus choroides, had not been long deposited there. The communication between the lateral ventricles, under the fornix, was large and distinct. There was pus lodged on the right side of the tentorium cerebelli; and the dura mater on the right temporal bone was detached, thickened, and diseased. The cerebellum was sound, but imbedded in thick pus, some ounces of which lay upon the dura mater, investing it, and consequently immediately in contact with its under surface. The adhesions of the dura mater to the foramen magnum were natural and firm; but the pus seemed to descend between that membrane and the medulla oblongata, which had lymph adhering to it, as had every part of the membrane described to have been in contact with pus: namely, the whole space outside the dura mater down to and over the pars petrosa of the temporal bone: and that which was inside the dura mater from the ulcers downward, along the right side of the falx to the tentorium, and extending on the inside of the dura mater, as far as the foramen magnum. It was a thick layer, not separable by simple ablution, but easily rubbed off with the fingers.

I now sawed out a piece from the bodies of the three lowermost lumbar lumbar vertebræ, when, as I had anticipated, a quantity of pus flowed out from between the medulla spinalis and its membraneous investment; and still more copiously on raising the upper part of the trunk. Here the inside of the tunic also evinced the appearance of adhering lymph. There was no vestige of fracture in the skull, but the diploë of part of the right parietal bone was darker coloured than natural, and was somewhat thickened, as if undergoing incipient caries. The tables were sound.

"*Remarks.*—What appear to me as peculiarities in this case are, the supervention of disease at so unusually late a period as twelve months after the infliction of a blow, which at the time seemed to have occasioned very little inconvenience; the symptoms of inflammation commencing immediately after the cessation of the discharge from the ear, and the ulceration of the brain so quickly succeeding to this change; but, most particularly, the intolerable pain occasioned by the insinuation of the matter between the medulla oblongata and its investing membranes, down to the very extremity of the spine, a symptom, which, as far as I recollect, has not been hitherto noticed, and which, in this case, has been clearly manifested by the dissection. The pus between the cerebellum and dura mater was in considerable quantity, and after having found its way thither, obtained an easy passage onward; for I cannot suppose it was secreted there.

"The pathology of this case is more interesting than any practical inductions that may be drawn from it; because I believe its cure was beyond the art of surgery. On this point, however, I would beg to speak with great diffidence. Having copied the case from the notes taken at the bed-side, and endeavoured simply to delineate the appearances as they presented themselves on dissection, I am desirous of leaving all comments and deductions to my more experienced brethren."

Mr. R. Bampfield, surgeon of the royal navy, in a very ingenious essay, has described an affection of the sight, usually called Nyctalopia or Night Blindness, but which he more properly terms Hemeralopia. He divides it into species, idiopathic and symptomatic. It is common in tropical climates, and if not combated by remedies, frequently terminates in total blindness. The author found that blisters applied to the temples, and repeated, if necessary, several times, in all cases effected a cure. The second species, however, depending on scorbutus, required that complaint to be removed, before it would yield to the remedy.

An interesting case is detailed by Dr. Clark, surgeon to the forces. He extracted three small cartilaginous substances from the cavity of the knee joint at three successive operations. The use of the joint was completely restored.

Dr. Bostock has described some experiments which he had an opportunity of making on the urine and serum of the blood blood of a young lady who had been taking large quantities of soda.

Mr. Chevalier, surgeon to the Westminster General Dispensary, has stated a case, in which the internal coat of the stomach and duodenum was lacerated by vomiting.

"—— Froome, aged 14, went out on Saturday, 25th of Dec. 1813, to a Christmas feast, ate heartily, and drank rather freely of gin and water; on the evening of the following day he became sick, and vomited violently. The vomiting continued, at intervals, during the whole of that night and the following morning. He went out, however, for a short time, but felt very ill; and said to one of his companions, that his blood was boiling at his heart, and that he thought he should die; and begged he would come to his funeral, if it were so. He soon returned home, and about two o'clock on Monday afternoon he became short breathed, unable to swallow, and felt great anxiety, with almost continued efforts to vomit. These symptoms increased till the following day, when my friend Mr. Lightfoot, of Oxford-street, saw him, and, thinking his situation dangerous, desired that I might be sent for.

"I visited him about noon; his appearance was extremely affecting; his countenance was flushed and turgid; his breathing anxious and interrupted; his pulse very irregular; and his extremities cold. He complained of great uneasiness at his heart, which was increased by pressure near that viscus, the action of which consisted in the successive repetition of three irregular strokes; the first rather violent, the second feeble, and the third still more so. His attempts to vomit were frequent and most distressing, and generally terminated in the discharge of white froth. When he attempted to drink, the effort to swallow was accompanied by a violent and agonizing spasm of the pharynx, which made him dash the cup from his hand. Pressure on the region of the stomach, particularly towards the right side, occasioned him a great increase of pain, and an immediate recurrence of the efforts to vomit. Towards the evening, he vomited in successive efforts near two pints of blood; after this he became easier, and said he should like to eat something; toasted bread was given him, of which he ate two pieces rather eagerly, but the vomiting speedily returning, he threw up what he had eaten, and soon after this a quantity of something which he said was so bitter it was enough to kill him. Almost immediately after this he expired.

"I opened the body the following morning. The left lung was found to adhere to the pleura very generally; in every other respect the thoracic viscera were healthy. The heart, and every thing connected with it, appeared entirely in a natural state. The viscera of the abdomen also appeared to be healthy externally; but, in laying open the stomach and duodenum, the internal coat of both appeared to be torn in various places. These lacerations were much larger in the duodenum than in the stomach; and near the pylorus they extended nearly round the circumference of the gut, and and rendered this part so weak that it was quite torn off in a very slight effort to loosen this portion of the intestine for the purpose of passing a ligature round it. Two extensive lacerations were also found near the middle of this portion of the intestine. All that remained of the intestinal tube, and also the whole of the œsophagus, and all the rest of the abdominal viscera, were perfectly free from any morbid appearance."

Mr. Henry Earle, surgeon to the Foundling Hospital, has communicated some original and ingenious observations on contractions after burns or extensive ulcerations. The mischief resulting from these accidents is obvious, and of frequent occurrence; we shall therefore be gratified to learn, on some future occasion, that the treatment proposed by Mr. Earle has proved successful in more cases than in the one which he has now related.

Dr. R. B. Cheston, Physician to the Gloucester Infirmary, has related the history of a child retained in the mother fifty-two years after the usual period of utero-gestation, so curious and extraordinary, that, though long, we shall present it to our readers in the words of the author. The case is illustrated by plates, but it is sufficiently intelligible without their addition.

"In the month of December, 1738, Mrs. Cowles was taken in labour with her fourth child, having gone her usual time without any circumstances differing from her former pregnancies; the pains were lingering, and went on for three days, but without any advances towards delivery, under the attendance of a female midwife, who had been with her on former occasions. The late Mr. Rogers, of Gloucester, an accoucheur of long established practice, and deserved eminence in his profession, was then called in, and, upon examination, declared that the child offered for the birth, but that he could not deliver it without instruments, as the pains were not sufficient to bring the child into the world. This kind of assistance Mrs. C. positively refused, under the idea that the child would be thereby sacrificed, and she firmly declared, that, if she could not be delivered without instruments, she and the child should die together.

"For some days the pains seemed to return at intervals, but gradually abated, so that by the end of the week all prospect of delivery was over. Great uneasiness still continued in her belly, confining her to the room, and she suffered much mental anxiety from her situation. As the case appeared so remarkable in many respects, Mr. Rogers was pressed for his sentiments on the probable termination of it, when he declared, that now the child would not be born, but that at some distant period, the bones of it would be found with the flesh gone. This account I received from Mrs. Cowles's sister, who lived constantly with her, attended at her labour, and was alive at the time of her death.

"In the year 1771, thirty-three years after her expected time of delivery, delivery, I was desired to meet Mrs. C. at the house of a very intelligent friend of hers, who had been fully acquainted with every circumstance of her former situation, and who then confirmed to me the foregoing relation.

"At this time Mrs. C. requested, that, after her death, I would satisfy myself concerning the real nature of her case, about which so many doubts had been raised.

"From herself I learned the following particulars:—

"In the third year after the birth of her last child, she considered herself again pregnant, and felt in every respect as in her former pregnancies. The motions of the child were lively; she had milk in her breasts; and, as her labour seemed to come on, she felt the same kind of pains as before, though in a slighter degree, and with less bearing down, or effort for the birth. The pains of child-birth left her about the third day, and she continued in a very weak distressed state for full three months afterwards, when she gradually recovered her strength, and from that time had found but little diminution in size.

"Upon inquiring particularly whether she ever afterwards menstruated, she informed me that she had had several appearances at different times, but they had not made a sufficient impression on her mind, to enable her to speak to such a circumstance with the necessary precision. She had frequently been sensible of a gentle motion within the abdomen, when she was in bed, but very seldom when she was up or walking about; never found any pressure upon her bladder, so as to excite particular inclination to make water; and that had it not been for her increased size, she had not felt any material cause for complaint. Her general health was very little affected, and to the last year of her life she employed herself busily with her family concerns.

"In her 80th year she had a slight paralytic stroke. In February 1790, I was desired to visit her, as her friends were then alarmed at the change in the appearance of an ulcer on the toe, which had been of some standing, but not particularly troublesome. I found her in bed, with a small fluttering pulse, cold and discoloured extremities, and every sign of quick-spreading mortification in the affected foot. Being desirous to ascertain the general state of the abdomen, I examined it carefully, and found a considerable emphysematous crackling within the cavity, but not the least tension; so that a perfectly circumscribed tumour could be felt about two fingers breadth higher than the navel, but inclining to the right side. She died in a few hours.

"*Dissection.*—The lymphatics of the affected leg and thigh, from the foot up to the groin, were more conspicuous from the inflammation they had undergone, than any I had ever before observed, the limb being entirely covered by a red net-work.

"Upon exposing the cavity of the abdomen, a tumour immediately presented itself, covered by the omentum and small intestines, which adhered to it firmly. When these attachments were separated, separated, it presented a complete bony surface, which yielded, upon striking it, the sound of a solid bone.

" As the supposed nature of the case called for a careful examination into the state of the uterus, I searched for it in its usual situation (for the tumour would admit of being raised from the sides and brim of the pelvis on which it rested), but could not distinguish it. Taking, therefore, the advantage of an examination per vaginam with one hand, while the other was in the pelvis, I at last met with a small firm substance, at the extremity of the vagina, lying flat at the bottom of the pelvis, but, as it had not the usual plump and hardened feel of the os tincæ, I became the more anxious to take out the whole contents of the pelvis, together with the tumour, that I might not injure any part of consequence to the grand object of my research. This I effected with no small trouble, and brought it away with me, for more minute examination at home. Extending my inquiries to the state of the other viscera, I found the liver of a very dusky hue, and particularly soft in its texture, but of its natural size. The gall-bladder had assumed the particular shape of a crescent, having both extremities filled with concretions of various sizes. There was likewise a quantity of thick viscid bile, which filled an ounce measure. The ducts were so large, that, upon pressure, the bile, as well as small calculi, readily passed through the ductus communis. The kidneys were natural in size and appearance. The spleen, like the liver, was soft, and deeply livid. The stomach and intestines were healthy.

" As, in the course of dissection, I found the greater arteries in a state of ossification, I took out the heart, with its immediate ascending branches, and the aorta descendens, as far as the iliacs within the abdomen, when the whole of these vessels proved more or less loaded with large deposits of earthy matter, particularly the emulgents, to their entrance into the kidneys, and the splenic artery even within the very substance of that viscus. The heart was soft and flaccid, without any other circumstance worth remarking, unless that the coronary arteries, for some length, were much hardened, but without any apparent deposit of earthy matter. The lungs, on the right side, adhered firmly and universally to the pleura; on the left, partially; but in general looked healthy.

" The adhesions of the tumour rendered the natural distinctions of what I had reserved for my examination at home rather obscure; but I at last ascertained the following circumstances. The upper part of the vagina was in a natural state, but the os tincæ appeared nearly as much reduced as at its dilation in a natural labour. Dividing, however, what was evidently the lower part of the uterus, I found its substance, though diminished in thickness, still retaining somewhat of its natural structure; its internal surface exhibited very clear remains of the plaited appearance which characterises the cervix, for about three inches, when it became contracted to an obtuse point, with an aperture which just admitted the round end of a probe to pass onwards in a strait direction. Upon laying this open, there appeared a sulcus strongly marked with rising sides for about two inches, when they diminished gradually, till they were almost obliterated at its termination, in a small round cavity just of a size to receive a full-grown pea.

"The spermatic vessels were very evident on the right side, attached to the superior part of the elongated uterus, but no trace of the ovarium could be discovered; and it was only after a very attentive search that I found a cord-like substance, about the size of a crow-quill, in its external appearance not unlike the vas deferens. Upon discovering that this was tubular, I slit it up, and found, by the elegantly plaited appearance of its internal surface, that it was undoubtedly the fallopian tube. On the left side, the fallopian tube, possessing in every respect its natural course and appearance, and terminating as usual in its fimbriated extremity, was very obvious. I could not, however, satisfactorily ascertain the existence of the ovarium. The close adhesions to the neighbouring parts, and the pressure occasioned by the tumour, will sufficiently account for this circumstance.

"On separating the soft parts, the bony surface was found perfectly complete, except at that point where the cavity terminated; and here, for about the size of a sixpence, the ossification was deficient, and the aperture filled up with a steatomatous or sebaceous matter, which readily suffered a probe to pass within the tumour, about half an inch, when it was prevented from going further by a firm resisting substance. The bony mass, now divested of every thing connected with it, very much resembled a human cranium of a middle size, but round rather than oblong, weighing, with its contents, three pounds, one ounce, four drachms.

"Though the circumstances first-mentioned were sufficient to support an opinion that a fœtus might be inclosed in this bony case, yet the possibility of its being an enlarged ovarium, or some substance of a glandular nature in a state of ossification, determined me to divide it through the middle with a fine amputating saw, which would but slightly derange its contents. If it proved to contain a fœtus, the divided parts might be readapted so as to show their natural connections, and such means pursued as would best assist any further inquiries.

"A longitudinal section was therefore carried through the middle of the bony mass, and the divided surfaces, on a superficial inspection, very much resembled the kind of tumour frequently met with in the different viscera, and composed apparently of cartilaginous layers, whose interstices are filled up with steatomatous matter. I soon, however, discovered that it consisted of an osseous cyst, containing the body of a full-grown fœtus, generally speaking in a state of wonderfully perfect preservation. The course of the saw had been very favourable to the subsequent examination of the parts. It had passed obliquely through the head and trunk in a direction from before backwards, commencing on the right side of the head, continued through the right shoulder, and the same side side of the chest, which cavity was just exposed; then slanting from the right hypochondrium through the middle of the pelvis. Of the extremities, the left leg only was cut through; one half of the tumour contained the greatest part of the head, with the whole of the neck, nearly the entire chest and abdomen, the left upper extremity, and the thigh and upper portion of the leg of the same side. In the other division, I had a slice of the right part of the face and chest, the whole right upper and lower part of the left leg, with the foot of the same side."

Want of room obliges us to defer the remainder of this case, which, with the following, will appear in our next.

---

